# Analysis of Mesoscopic Parameters of Porous Asphalt Concrete and Its Impact on Permeability Performance

**DOI:** 10.3390/ma18133062

**Published:** 2025-06-27

**Authors:** Qiuming Zhou, Chupeng Chen, Pengguang Liu, Zebang Deng, Fucheng Guo, Dingbang Wei

**Affiliations:** 1 Guangdong Road and Bridge Construction Development Co., Ltd., Guangzhou 510635, China; 2 Guangdong Hualu Transport Technology Co., Ltd., Guangzhou 510550, China; 1112216034@e.gzhu.edu.cn; 3 Gansu Industry Technology Center of Transportation Construction Materials Research and Application, Lanzhou Jiaotong University, Lanzhou 730070, China; 4 Gansu Transportation Planning, Survey and Design Institute Co., Ltd., Lanzhou 730030, China

**Keywords:** porous asphalt concrete, mesoscopic parameters, permeability performance, CT scanning, CFD simulation

## Abstract

Porous asphalt concrete (PAC) is widely recognized for its excellent performance in drainage, noise reduction, and environmental protection due to its high interconnected porosity. However, challenges remain in relationships between mesoscopic void parameters and permeability performance. To reveal the influence mechanism of meso-structural parameters on the permeability performance of PAC, the X-ray CT scanning and computational fluid dynamics (CFD) simulation techniques were combined in this study. A PAC-13 mixture was selected and prepared with target porosities of 18%, 20%, and 25%. The three-dimensional meso-structure of the specimens was measured using a CT scanner with a resolution of 0.08 mm, and the void parameters were extracted using Image J v1.8.0 software. The mesoscopic parameters of PAC and its impact on permeability performance were analyzed. Moreover, a three-dimensional void model was reconstructed using Avizo 9.0 software. The seepage performance was analyzed using CFD simulation. The results show that the roundness, the ratio of long to short axes, and the equivalent diameter of the voids increase linearly with porosity from 18% to 25%. The void number distribution shows a Gaussian characteristic. The permeability coefficient of PAC mixtures gradually increases linearly with the increase in porosity from 18% to 25%. Good relationships can be found between mesoscopic distribution characteristics and the permeability coefficient, where the coefficients of determination are larger than 0.97. The surface seepage pressure is nearly ten times more than the bottom pressure. The influence depth of seepage pressure is deeper with the increase in porosity, while the seepage velocity increases with the increase in porosity. This study offers valuable insights into the functional design and performance optimization of PAC materials.

## 1. Introduction

Porous asphalt concrete (PAC), an environmentally friendly pavement material with a high interconnected porosity, has been widely considered in the field of road engineering in recent years due to its excellent drainage, noise reduction, and anti-skid performances. Compared with traditional dense-graded asphalt mixtures, the porosity of PAC usually ranges from 15% to 25%. The seepage channels formed by its internal interconnected voids can quickly drain surface water, significantly improving driving safety and comfort [1,2,3]. In addition, the void structure of PAC can effectively absorb the noise generated by the contact between tires and the pavement, with a noise reduction effect of 3–5 dB (A) [4,5]. In terms of environmental protection, PAC mitigates the urban heat island effect and replenishes groundwater resources by reducing surface runoff and promoting rainwater infiltration [6]. However, the high porosity leads to relatively weak mechanical properties in PAC, so balancing its durability and functionality has become a core challenge in design and application. Therefore, an in-depth investigation of the mesoscopic regulation mechanism of PAC material properties is of great significance for optimizing its design and extending its service life.

The macroscopic water permeability and durability of PAC are directly influenced by its mesoscopic structural parameters, such as void shape, connectivity, equivalent diameter, and distribution characteristics. Existing studies have mainly focused on the correlations between macroscopic parameters (e.g., porosity, gradation, asphalt content) and performance, and predict drainage capacity based on empirical formulas or statistical models [7,8,9]. However, macroscopic parameters cannot accurately characterize the heterogeneity and complexity of the void structure, leading to limited prediction accuracy of the models. For example, PAC specimens with the same porosity may exhibit significantly different permeability coefficients due to differences in void connectivity [10]. In recent years, researchers have gradually recognized the decisive role of mesoscopic parameters in seepage behavior. Nevertheless, restricted by the resolution of traditional testing methods, related studies are still dominated by two-dimensional cross-sectional analyses or simplified models, making it difficult to establish a quantitative relationship between mesoscopic structure and macroscopic performance [11]. Therefore, it is urgent to develop high-precision mesoscopic characterization methods and reveal the dynamic influence laws of mesoscopic parameters on the seepage mechanism.

The rapid development of Computed Tomography (CT) technology has provided a new approach for the three-dimensional non-destructive characterization of the mesoscopic structure of materials. High-resolution CT scanning can acquire parameters such as the morphology, distribution, and connectivity of internal voids in porous asphalt concrete (PAC), and digital image processing techniques (e.g., threshold segmentation, fractal dimension calculation) can be used to achieve a quantitative analysis. For example, Li et al. [12] found through CT reconstruction that the distribution characteristics of equivalent void diameters exhibit a nonlinear relationship with the permeability coefficient. Meanwhile, computational fluid dynamics (CFD) simulation technology can analyze the flow behavior of fluids in complex void networks [13]. By coupling the Navier–Stokes equations with porous medium models, the effects of mesoscopic parameters (such as void roundness and fractal dimension) on seepage velocity and pressure field can be quantified [14,15,16,17]. However, existing studies mostly use idealized geometric models or local slice data and fail to conduct full-scale seepage simulations combined with real three-dimensional void structures. Therefore, integrating CT scanning and CFD simulation technologies to construct a seepage model based on real mesoscopic structures is crucial to break through theoretical bottlenecks [18,19,20,21].

In this study, a PAC-13 mixture was selected as the research object to systematically investigate the influence mechanism of mesoscopic parameters on the drainage performance of PAC. First, PAC specimens with different target porosities were prepared by optimizing gradation design and asphalt content. Second, three-dimensional mesoscopic structures of the specimens were obtained using X-ray CT scanning technology, and parameters such as void shape, number distribution, and equivalent diameter were extracted. Finally, a real void reconstruction model was established based on computational fluid dynamics (CFD) simulation technology to analyze the distribution laws of seepage pressure and velocity fields, thereby revealing the regulatory mechanism of mesoscopic parameters on macroscopic drainage performance. The research results can provide a theoretical basis for the functional design and performance optimization of PAC materials.

## 2. Materials and Methods

### 2.1. Materials

#### 2.1.1. TPS High-Viscosity Modifier

PAC is a typical open-graded asphalt mixture with a high proportion of coarse aggregates and a low proportion of fine aggregates. Therefore, high-viscosity modifiers are generally used to improve the bond strength and anti-rutting capability of the asphalt mixture. In this study, TPS (TAFPACK-Super), an asphalt modifier specifically developed by Daido Construction Co., Ltd. from Nagoya in Japan for PAC pavements, was adopted. Its main component is thermoplastic rubber, with other components including adhesive resin and plasticizer. It appears as a yellow granular elastomer with a particle size of 2–3 mm, a specific gravity of 0.98, and a density of 600 kg/m^3^.

#### 2.1.2. Asphalt Binder

Due to the large content of coarse aggregates in PAC, the asphalt film on the surface of aggregate particles is relatively thin, and the aging resistance performance might be inadequate when the base asphalt binder is used. It is necessary to use a high-viscosity asphalt binder to produce sufficient constraints and restrictions on the skeleton structure of coarse aggregates to improve the mechanical properties of the asphalt mixture. In this study, an SK70 base asphalt binder, manufactured by SK HOLDINGS in Seoul of Republic of Korea was selected, and a TPS high-viscosity additive produced by Dayou Construction Co., Ltd. of Nishihara, Japan was used. The dosage ratio of the TPS and base asphalt binder was 12:88. The technical properties of the TPS-modified high-viscosity asphalt binder are shown in Table 1. Moreover, the technical properties of the asphalt binder were also measured after the Rolling Thin Film Oven Test (RTFOT), a method to simulate long-term aging. As shown in Table 2, all technical properties met the requirements of the specification JTG E20-2011 [22].

#### 2.1.3. Coarse Aggregate

In this study, the high-quality basalt produced by Yongdeng Jianxin Stone Factory in Lanzhou City, in China was selected, and the basic properties of the coarse aggregate were tested according to the test specification of JTG E42-2005 [23]. All performances met the requirements.

#### 2.1.4. Fine Aggregate

In this study, the manufactured sand produced by Yongdeng Jianxin Stone Factory in Lanzhou City, China was selected, and the basic properties of the fine aggregate were tested according to the test specification of JTG E42-2005. All performances met the requirements.

#### 2.1.5. Mineral Fines

In this study, limestone mineral powder was selected. The basic properties of the mineral powder were measured according to the test specification of JTG E42-2005. All performances met the requirements.

### 2.2. Specimen Preparation

#### 2.2.1. Design of Gradation

In this study, mixed PAC-13 was selected. The porosity of PAC needs to be balanced between drainage performance and durability. Previous studies have shown that the recommended porosity range is between 18% and 25% [24]. In this study, three different porosities were selected as 18%, 20%, and 25%, respectively.

**Figure 1:**
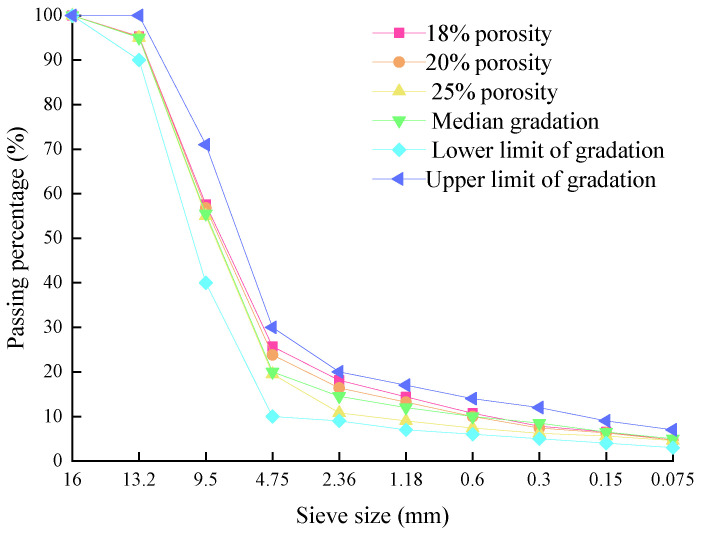
Gradation curves of PAC 13 with different target porosity.

The proper control indicators should be selected to ensure that the designed graduation can meet the required porosity. Previous studies have shown that the porosity of PAC has a significant linear correlation with a 2.36 mm sieve pass rate, indicating that the target porosity can be accurately controlled by adjusting the sieve pass rate. The gradation of each target porosity for PAC-13 is determined using this control method, as shown in Figure 1.

#### 2.2.2. Determination of Optimum Asphalt Content

The optimum asphalt content of other PAC mixtures in this study was determined according to the Marshall design method. The optimum asphalt content of PAC-13 mixture with each target porosity is shown in Table 2. The Marshall specimens of PAC-13 with different porosities were prepared using the designed gradation and optimum asphalt content.

### 2.3. Test Methods

#### 2.3.1. X-Ray CT Scanning and Digital Image Processing Technique

X-ray CT scans the detected object by transmitting an X-ray beam, and the X-ray transmitted through the object is received by the detector at the other end. The detector converts the received signal to analog-to-digital conversion and is further processed by the computer to obtain a CT image [25]. Asphalt mixture is a mixture of asphalt mortar, aggregate, and voids. Different materials have a different absorption and attenuation of X-rays, and their internal structure can be obtained by CT scanning technology. In this study, a Y. CT Precision II high-energy high-resolution industrial CT scanner was adopted to capture the three-dimensional geometric structure of mixture specimens, where the scanning voltage of 210 kV, a current of 0.36 mA, and a scanning resolution of 0.08 mm were adopted. The tomography images of PAC were obtained with the interval of 0.01 cm from top-to-bottom.

The original image obtained by CT scanning has the problems of noise and uneven brightness, which needs to be optimized and analyzed by digital image processing (DIP) [26]. The principle of DIP technology is to use a series of algorithms to process and analyze the digital matrix in the image by computer. In this study, the image processing function of Image J v1.8.0 was used to post-process the CT scan images of PAC specimens, and then the void parameters were extracted and analyzed.

**Figure 2:**
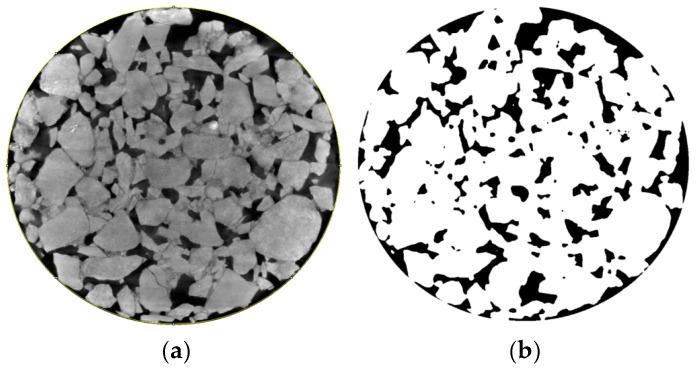
Image threshold segmentation results: (**a**) original drawing; (**b**) binary picture.

DPC technology is a complex process, mainly including image enhancement and image segmentation. During the image enhancement process, gray-scale treatment, image denoising, and histogram equalization were conducted. Gray-scale treatment aims to convert the color image into an 8-bit grayscale image with a gray level range of 0–255. Furthermore, Gaussian filtering was used to filter the noise in CT images. Histogram equalization was conducted to change the histogram of the original digital image into a form of uniform distribution in the whole gray range by pixel gray value conversion. Image segmentation refers to the process of accurately extracting the part of the image that needs to be analyzed. This is the premise of accurately obtaining image information. The key to image segmentation is to determine the threshold. Suppose that the image is divided into two parts: the background and foreground. Entropy represents the amount of information. The maximum entropy algorithm finds an optimal threshold to maximize the sum of the entropy of the two parts of the background and the foreground. On the basis of using the maximum entropy method to determine the segmentation threshold, this study fine-tunes it appropriately by comparing it with the original image [27]. The result of image segmentation is shown in Figure 2.

Comparing Figure 2a,b, it can be seen that the maximum entropy method can be used to segment the void information completely. The different void geometries can be clearly characterized in the segmented image. In Figure 2b, black represents the void and white represents the aggregate. Therefore, in the subsequent experiments, the maximum entropy method was used to segment the CT images of PAC specimens and perform related calculations. The distribution characteristics of mesoscopic parameters were analyzed according to the images after CT scanning and image processing.

#### 2.3.2. Evaluation of Mesoscopic Parameters of Mixture Void

(1) Void shape

The shape of voids is a key factor affecting the permeability of porous materials. The parameters used to characterize the void shape are roundness, a long and short axis ratio, etc. Void roundness refers to the degree to which the shape of the void cross section is close to the theoretical circle. The calculation formula of the void roundness is shown in Equation (1):

(1) $$R=\frac{\sum_{i=0}^{n}\frac{L_{i}^{2}}{4\pi A_{i}}}{n}$$

where *R* is the void roundness of the PAC specimen; *L* is the total perimeter of the void of a section, mm; *A* is the total void area of the section, mm^2^; and *i* is the section number.

The ratio of long axis to short axis indicates the degree of the equivalent void ellipse approaching the theoretical circle. Similar to roundness, the void will be narrower with the larger ratio of long axis to short axis. The calculation formula of the ratio of long to short axes is shown in Equation (2).

(2) $$AR=\frac{\sum_{i=0}^{n}\frac{R_{li}}{R_{Si}}}{n}$$

where *AR* is the ratio of long to short axes of the PAC specimen; *R_l_* is the long axis of the equivalent ellipse of the section void, mm; *R_S_* is the short axis of the equivalent ellipse of the section void, mm; *i* is the section number.

(2) Number distribution of voids

The number of cross-section voids *n* is the sum of the number of voids in each section obtained by Image J statistics after the threshold processing of CT images of each section of a single specimen.

(3) Equivalent void diameter distribution

The average equivalent void diameter *d* of the section is the total void area divided by the number of voids after the threshold processing of the CT original photos of each section of a single specimen, and then the diameter of a single void is obtained after the weighted average. The calculation formula is as shown in Equations (3)–(5).

(3) $$A=\frac{\sum_{i=1}^{n}A_{i}}{n}$$

(4) $$d=2\sqrt{\frac{A}{\pi }}$$

(5) $$d\prime =2\sqrt{\frac{A_{i}}{\pi }}$$

where *A* is the average void area of a section, mm^2^; *A_i_* is the area of any void on a section, mm^2^; *n* is the number of voids; *d* is the average equivalent void diameter of the section, mm; and *d’* represents the equivalent diameter of any void in the section, mm.

#### 2.3.3. Permeability Coefficient Test

**Figure 3:**
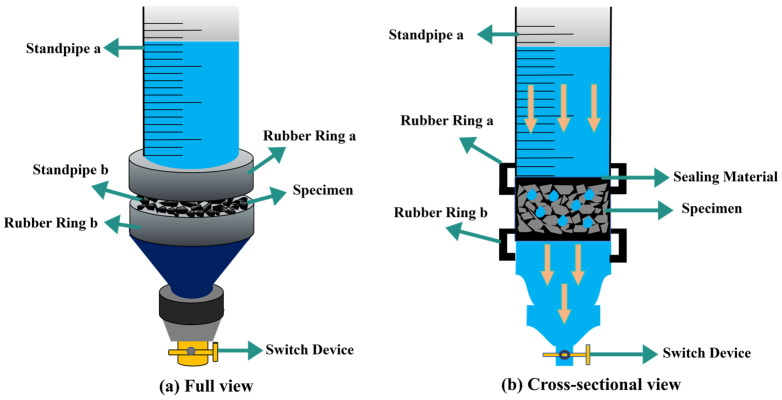
Schematic diagram of permeability coefficient test device.

The permeability coefficient has been widely used to characterize the permeability of porous asphalt pavements. In this study, a self-developed permeability testing device was used to measure permeability, and the schematic diagram is shown in Figure 3. The detailed information related to the device can be seen in the previous publication [24].

#### 2.3.4. CFD Penetration Simulation Technology

Computational fluid dynamics (CFD) is a calculation method based on mass conservation equation and momentum conservation equation [19]. It is used to simulate the change of fluid flow field in the void and obtain the flow field data in the post-processing. This is the most widely used fluid simulation method.

**Figure 4:**
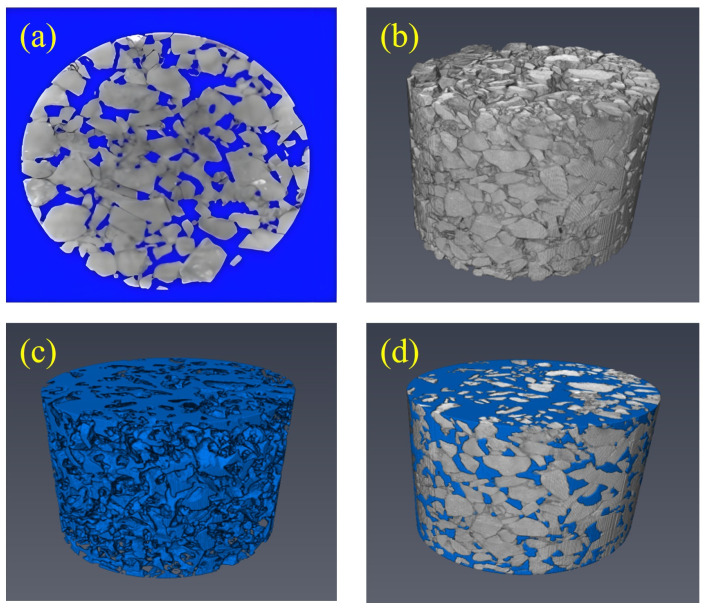
Three-dimensional reconstruction process of PAC specimen void: (**a**) Binary segmentation; (**b**) skeleton model; (**c**) 3D void structure; (**d**) skeleton and void diagram.

In this study, the CT scan images obtained in the second part of the study were used for a three-dimensional reconstruction of the void. Due to objective reasons such as specimen forming and CT scanning, it is inevitable that the scanning effect of the top and bottom will be poor and the imaging blurred in the scanning. Therefore, three pictures before and after the elimination were removed. Avizo 9.0 image processing software was used in this study to process CT images and achieve 3D reconstruction. The information from 3D images, void geometry models, or numerical simulation data can be directly extracted and analyzed using this software. The three-dimensional reconstruction model was generated by using median filtering, volume editing, and binary image segmentation. The processed skeleton void model is shown in Figure 4, where the gray part of the image is the skeleton, and the dark blue marker area inside the skeleton is the void.

The internal void structure of the specimen is composed of closed void, semi-connected void, and connected void. In the actual seepage, only the connected void is the key factor to determine the seepage capacity, while the non-connected void and semi-connected void will not affect the seepage performance, but the semi-connected void will affect the seepage process. To ensure the quality of the void model after three-dimensional reconstruction, the disconnected voids can be removed to generate a three-dimensional void model that retains the effective voids.

From the results of the CT scan, the number of voids in PAC specimens is large and the void structure is complex. Therefore, part of the void structure is intercepted in the three-dimensional reconstruction model for numerical simulation. The volume of connected voids in the three-dimensional reconstruction model of PAC specimens increases with the increase in porosity.

To verify the accuracy of the model built by Avizo, the void fraction of the three-dimensional reconstruction model was calculated and compared with the experimental values. The effective porosity of the three different porosity specimens was calculated as shown in Table 3.

It can be seen from Table 3 that the absolute difference between the experimental value and the calculated value of the three porosity specimens is between 0.3% and 0.7%, and the error is between 1.5% and 3.8%, indicating that the three-dimensional reconstruction model is very accurate in calculating the porosity.

**Figure 5:**
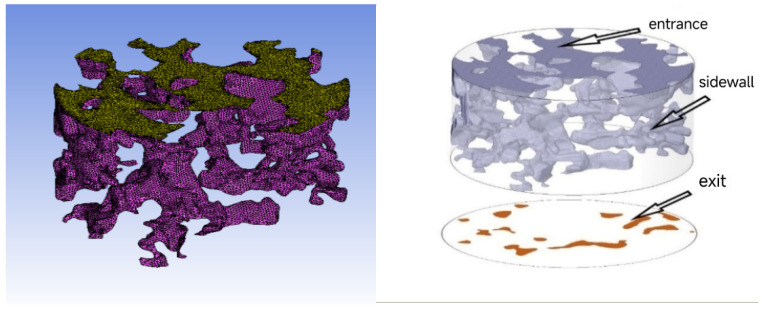
Model mesh division and void boundary condition setting.

In this study, the CFD simulation of the seepage flow of the three-dimensional void reconstruction model of the PAC specimen is mainly based on the Fluent 17.0 commercial software based on grid calculation and the open source OpenFOAM 11 and other fluid calculation software. The model was smoothed, geometrically cleaned, boundary marked, and meshed. The mesh quality affects computational efficiency and simulation accuracy. The mesh densities were finally determined as 3 million cells with the low convergence, which was also verified by the previous study [28]. Finally, the generated mesh file is imported into Fluent for numerical simulation. According to the actual test conditions, the internal flow field simulation of the model is carried out with 1000~3000 Pa as the simulated water pressure in the simulation. The PAC-13 specimen was selected, and the boundary conditions were set as shown in Figure 5.

## 3. Results and Discussions

### 3.1. Analysis of Mesoscopic Parameters of Voids Based on X-Ray CT

#### 3.1.1. Analysis of Void Shape

**Figure 6:**
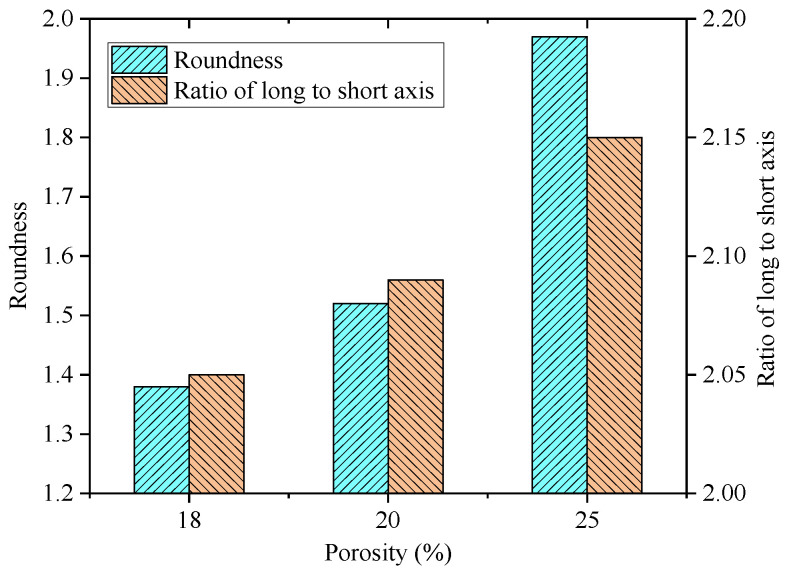
Variations of the roundness and ratio of long to short axes for PAC with different porosities.

In order to investigate the variations of void shapes in PAC specimens with different porosities, the roundness and the ratio of long to short axes were calculated, as shown in Figure 6.

As can be seen from Figure 6, the void roundness of PAC specimens was greater than 1, with an overall distribution ranging from 1.3 to 2.0, indicating the absence or minimal presence of “circular” voids within the PAC specimens. The void roundness increases to varying degrees as the porosity increases. Compared to PAC specimens with a porosity of 18%, the mean increase in roundness for specimens with porosities of 20% and 25% was 15.5% and 58%, respectively, indicating a greater increase in roundness with a higher porosity. This may be attributed to the fact that specimens with a higher porosity contain a larger proportion of coarse aggregates, and the connected pores formed by coarse aggregates are less filled with fine aggregates, resulting in void cross-sections that are closer to circular shapes. Similarly, the variation range of the ratio of long to short axes of voids was between 2.02 and 2.15 with the increase in porosity, showing an increasing trend but with overall little change, indicating that the internal void shape characteristics of the PAC mixture were predominantly elongated and narrow.

#### 3.1.2. Number Distribution of Voids

**Figure 7:**
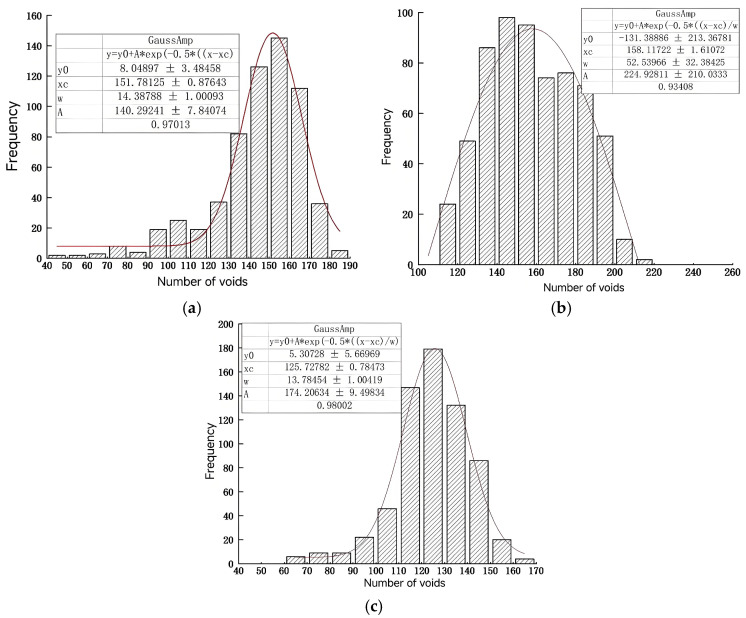
Void number distribution of PAC-13 specimen with different porosities: (**a**) 18% porosity; (**b**) 20% porosity; (**c**) 25% porosity.

To further clarify the number distribution of voids in different PAC specimens, the number of voids in CT tomography images of the PAC specimens with different porosities was statistically analyzed, and the Gaussian distribution curve was fitted. The results are shown in Figure 7.

From Figure 7, the number distribution of voids in PAC-13 specimens was Gaussian. For the specimens with porosities of 18%, 20%, and 25%, the distribution ranges of the number of voids were 40~190, 100~220, and 60~170, respectively. This shows that the number of voids in different porosity specimens spans differently, and the span gradually decreases with an increase in porosity. In addition, compared with Figure 7a–c, the number of voids in PAC specimens with target porosities of 18%, 20%, and 25% was concentrated within 130~170, 130~190, and 110~150, respectively. This shows that as the porosity increases, the peak value of the number of voids gradually decreases; that is, the larger the porosity, the less the number of voids. This is mainly because the PAC mixture with the larger porosity has more coarse aggregate content and less fine aggregate content, which makes it easy to form connected large voids.

The distribution of the number of voids above conforms to the Gaussian distribution [29]. Gaussian distribution is a very important probability distribution in the fields of mathematics, physics, and engineering, and has great influence in many aspects of statistics. If the random variable X obeys a Gaussian distribution with a mathematical expectation of μ and a standard deviation of σ, it is denoted by Equation (6).

*X*∼*N*(*μ*,*σ*^2^) (6)

Then the probability density function is as in Equation (7).

(7) $$f\left(x\right)=\frac{1}{\sigma \sqrt{2\pi }}e^{-\frac{{\left(x-\mu \right)}^{2}}{2\sigma^{2}}}$$

The expected value μ of the Gaussian distribution determines its location, and its standard deviation σ determines the magnitude of the distribution. Regarding the distribution law of the area under the Gaussian curve, if the standard deviation is used as the measurement unit, the area under the normal distribution curve is 68.27% of the total area in the positive and negative 1 standard deviation, that is, in the (μ − σ, μ + σ) interval. The area is 95.44% in the range of the positive and negative two standard deviations, namely (μ − 2σ, μ + 2σ).

The number of voids is nonlinearly fitted, and the statistical Gaussian probability distribution parameters are shown in Table 4. Therefore, the number of voids for PAC with different porosities can be directly predicted using the built model.

#### 3.1.3. Equivalent Void Diameter Distribution

**Figure 8:**
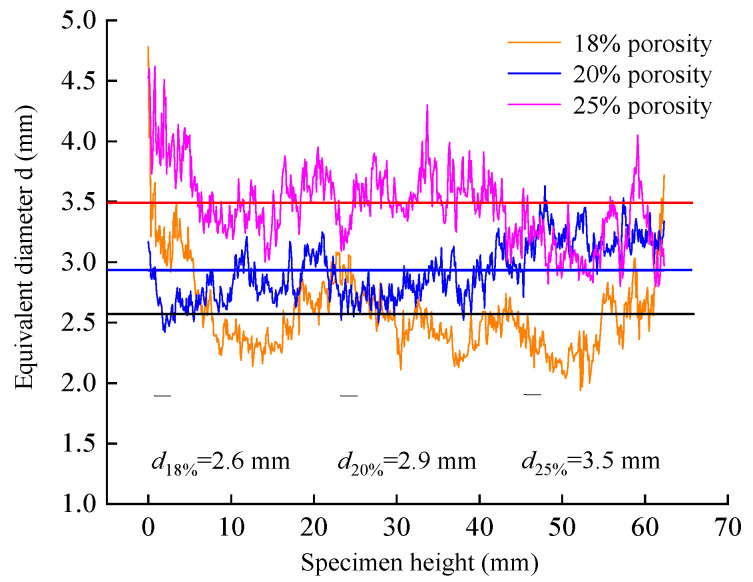
Variation curve of equivalent void diameters of PAC specimens with different porosities along the height of the specimen.

In order to study the distribution law of the equivalent diameter of PAC specimens, different equivalent diameters of different porosity are taken for analysis. Figure 8 is the change curve of the equivalent diameter of PAC specimens with different porosity along the height of the specimen.

It can be seen from Figure 8 that the equivalent diameter of the PAC specimen is consistent with the change trend of the porosity along the height of the specimen. The equivalent diameters of the PAC specimens with porosities of 18%, 20%, and 25% were 2.6 mm, 2.9 mm, and 3.5 mm, respectively, indicating that the equivalent diameter increases with the increase in porosity. The large equivalent diameter may induce the large pervious capacity and further enhance the permeable performance, which also proves that improving the porosity is an important means to improve the permeability of pavement. However, the large porosity will injure the durability and weather resistance. The relationship between mesoscopic parameters and drainage performance should be investigated further.

### 3.2. Effect of Mesoscopic Parameters on Permeability Performance

**Figure 9:**
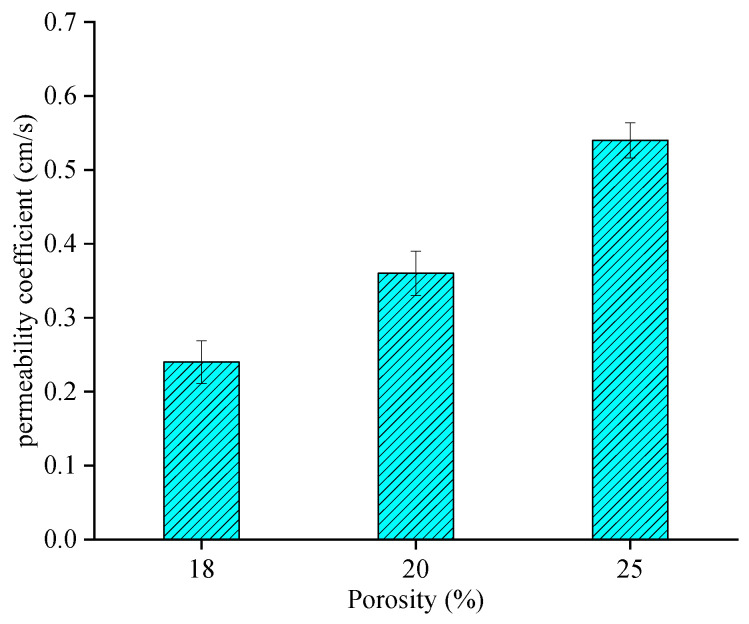
Permeability coefficient of PAC-13 with different porosities.

The permeability coefficient was measured for PAC-13 with different porosities, and the results are shown in Figure 9.

As shown in Figure 9, the permeability coefficient of PAC mixtures gradually increases linearly with the increase in porosity from 18% to 25%, which is consistent with the previous studies [30]. For the PAC-13 mixture, the permeability coefficients of mixtures with porosities of 20% and 25% were 50% and 125% higher than that of the mixture with a porosity of 18%. This indicates that the porosity has a significant effect on the permeability coefficient, which is consistent with conventional cognition.

**Figure 10:**
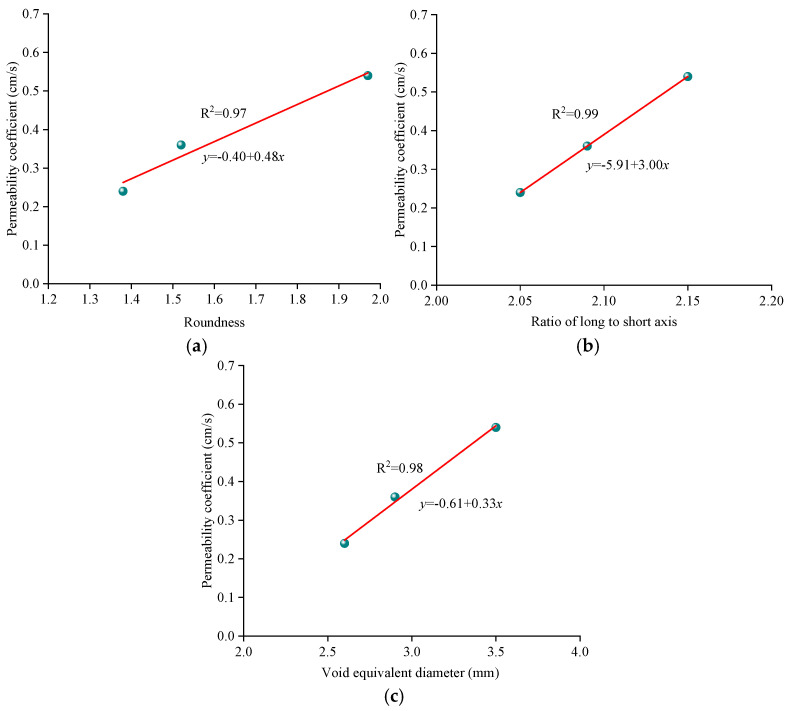
Relationships between mesoscopic void parameters and permeability coefficient: (**a**) roundness; (**b**) ratio of long to short axes; (**c**) equivalent void diameter.

From the previous analysis, the good correlations between mesoscopic void parameters and the permeability coefficient can be found, namely, the permeability coefficient increases with the roundness, the ratio of long to short axes, and equivalent void diameter. To quantitatively characterize the relationship, linear regression was conducted, and the results are shown in Figure 10.

As shown in Figure 10, good relationships can be found between mesoscopic void parameters and the permeability coefficient. The coefficients of determination in the linear regression models for three mesoscopic parameters were 0.97, 0.99, and 0.98, respectively. A good relationship between the permeability coefficient and roundness can be easily obtained using the principles of fluid mechanics. The larger equivalent void diameter is prone to permeating a higher volume of water through the specimen in the specific time. The favorable linear correlation between the equivalent void diameter and porosity has also been verified by Jiang et al. [31]. The results can explain the positive impact mechanism of porosity on the permeability coefficient at a mesoscopic level. Moreover, the conclusions also help to more accurately regulate the permeability of asphalt pavement from the perspective of microscopic parameters.

### 3.3. Numerical Simulation Analysis of Seepage Performance

#### 3.3.1. Analysis of Seepage Pressure of Different PAC Models

**Figure 11:**
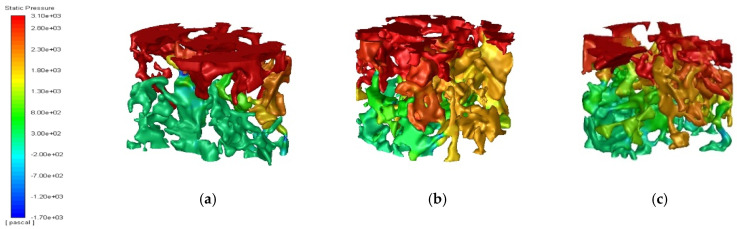
Seepage pressure of PAC reconstruction model with different porosity: (**a**) 18% porosity; (**b**) 20% porosity; (**c**) 25% porosity.

The CFD-PFC coupling method was used to analyze the seepage pressure of PAC-13 specimens under different porosities (18%, 20% and 25%). The seepage pressure cloud diagram is shown in Figure 11.

It can be seen from Figure 11 that the influence depth of seepage pressure was deeper with the increase in porosity. Moreover, the seepage pressure changed significantly with the specimen depth, where the surface seepage pressure was nearly ten times that of the bottom pressure. The penetration pressure depth of the 18% porosity model was significantly smaller than that of the 20% and 25% porosity models. From Figure 11b,c, it can be found that the seepage pressure of some voids can even directly reach to the bottom of the model, indicating that the number of connected voids was large, or the diameter of connected voids increased, which made the seepage flow increase, and the pressure of water flow on the void wall also increased.

#### 3.3.2. Seepage Velocity Analysis of Different PAC Models

**Figure 12:**
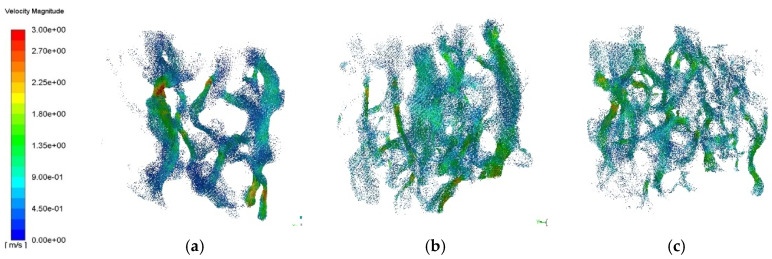
Seepage velocity diagram of PAC-13 reconstruction model with different porosities: (**a**) 18% porosity; (**b**) 20% porosity; (**c**) 25% porosity.

To analyze the seepage velocity of the PAC three-dimensional reconstruction model under different porosities, the seepage velocity diagram of PAC-13 specimens with 18%, 20%, and 25% porosities is shown in Figure 12.

From Figure 12, it can be seen more clearly that the seepage velocity increased with an increase in porosity, and the distribution of seepage velocity traces was greater, indicating that there were more seepage channels. In the 18% porosity model, two to three coarse seepage traces were formed. With the increase in porosity, the seepage traces increased rapidly and gradually became finer. When the porosity reached 25%, the seepage velocity increased slightly, and the seepage traces covered the whole model. This shows that as the porosity increases, the connected voids increase, and the void diameter also increases, resulting in a rapid increase in the seepage channel, and the water flow can pass through the model from multiple channels. As the porosity increases from 18% to 25%, the distribution range of the high seepage velocity area does not show a single increasing trend. Because the increase in porosity improves the connectivity, the void shape (such as the distribution of narrow and long channels) also affects the seepage velocity, indicating that the seepage velocity is not only controlled by the single factor of porosity, but is also related to the void structure.

## 4. Conclusions

The mesoscopic void is tightly related to permeability performance, while the specific relationship between mesoscopic void parameters and permeability performance still needs further analysis. In this study, the mesoscopic parameters of porous asphalt concrete (PAC), namely void shape, number distribution, and equivalent diameter, and its impact on permeability performance were systematically explored by the combination of CT scanning technology and CFD numerical simulation. The internal relationships among porosity, void structure characteristics, and permeability behavior were revealed, which provided a theoretical basis for optimizing the design and performance evaluation of drainage asphalt pavement. Based on the test results and simulation analysis, the following conclusions can be drawn.

(1) Using image filtering, image segmentation, and other image processing methods in Image J software, the meso-void information of PAC specimen CT images can be accurately extracted. The roundness, the ratio of long to short axes, and the equivalent diameter of voids increase linearly with the increase in porosity. The void number distribution shows a Gaussian characteristic; therefore, the void number distribution of PAC with different porosities can be directly predicted using the built model.
(2) The permeability coefficient of PAC mixtures gradually increases linearly with the increase in porosity from 18% to 25%. For the PAC-13 mixture, the permeability coefficients of mixtures with porosities of 20% and 25% are 50% and 125% higher than that of the mixture with a porosity of 18%. Good relationships can be found between mesoscopic distribution characteristics and the permeability coefficient, where the coefficients of determination of linear regression models for three mesoscopic parameters are 0.97, 0.99, and 0.98, respectively.
(3) The seepage pressure changes significantly with the specimen depth, where the surface seepage pressure is nearly ten times that of the bottom pressure. The depth of seepage pressure is deeper with an increase in porosity. The penetration pressure depth of the 18% porosity model is significantly smaller than that of the 20% and 25% porosity models. The seepage velocity increases with an increase in porosity from 18% to 25%, and the distribution channels of seepage velocity are abundant, resulting in a larger permeability performance.

## Figures and Tables

**Table 1:** Technical properties of high-viscosity modified asphalt binder.

Technical Properties		Unit	Measured Values	Requirements
Needle penetration (25 °C, 100 g, 5 s)		0.1 mm	49.5	≥40
Softening point (R&B)		°C	93.9	≥80
Ductility (15 °C)		cm	>100	≥50
Dynamic viscosity (60 °C)		Pa·s	496,731	≥20,000
RTFOF (163 °C, 85 min)	Mass variation	%	0.07	±0.6
	Residual penetration ratio (25 °C)	%	76.2	≥65
	Residual ductility (5 °C)	cm	29.1	≥6

**Table 2:** The optimum asphalt contents of PAC-13 mixture with different porosity.

Porosity	18%	20%	25%
PAC-13	5.1	4.9	4.5

**Table 3:** The effective porosity was calculated by PAC-13 three-dimensional reconstruction model.

Porosity (%)	Experimental Value (%)	Calculated Value (%)	Difference (%)
18	18.6	19.3	+0.7
20	19.7	20.0	+0.3
25	23.8	24.2	+0.4

**Table 4:** Fitting parameters of Gaussian distribution curve of voids number.

Porosity	18%		20%		25%	
Fitting parameters	*μ*	*σ*	*μ*	*σ*	*μ*	*σ*
Fitting values	158	52	151	14	125	14

## Data Availability

The original contributions presented in the study are included in the article; further inquiries can be directed to the corresponding author.

## References

[1] Zhong K., Fan J., Huang X., Chen M. (2022). Discrete element simulation on anti-rutting performance of PAC-13 pavement in urban roads. Mater. Struct..

[2] Darshan N., Kataware A.V. (2024). Review on porous asphalt pavements: A comprehensive resolution for stormwater management and applications in current built environment. Int. J. Pavement Res. Technol..

[3] McGhee P.E., Kevin K., Clark T.M. (2010). Functionally optimized wearing course: Installation report. Transp. Res. Rec..

[4] Jiang W., Lu R., Qing C., Li B., Zhou Y. (2023). Clogging characteristics and recovery effects of porous asphalt concrete with different gradations. Int. J. Pavement Eng..

[5] Margorínová M., Trojanová M., Decký M., Remišová E. (2018). Noise costs from road transport. Civ. Environ. Eng..

[6] Kou C., Qi Y., Kang A., Liu Y., Zhang L. (2021). Spatiotemporal distribution characteristics of runoff-pollutants from three types of urban pavements. J. Clean. Prod..

[7] Yu B., Sun Z., Qi L. (2021). Freeze-thaw splitting strength analysis of PAC based on the gray-Markov model. Adv. Mater. Sci. Eng..

[8] Montes F., Haselbach L. (2006). Measuring hydraulic conductivity in pervious concrete. Environ. Eng. Sci..

[9] Martin W.D. (2013). Influence of aggregate gradation on clogging characteristics of porous asphalt mixtures. J. Mater. Civ. Eng..

[10] Wang B., Zhang Y., Zhu X., Yao T., Li J. (2023). Experiment investigation and influence evaluation of permeability ability attenuation for porous asphalt concrete under repeated clogging conditions. Buildings.

[11] Liu Y. (2023). Study on the application of permeable pavement materials in road rehabilitation. Advances in Civil Engineering and Environmental Engineering, Volume 1.

[12] Li B., Sun M., Zhu X., Yao T., Guo F. (2023). Investigation of permeability persistence of porous asphalt concrete under coupled conditions of clogging and cleaning. J. Transp. Eng. Part B Pavements.

[13] Kong J., Jeong S., Lee J., Kim H. (2025). Permeable pavement blocks as a sustainable solution for managing microplastic pollution in urban stormwater. Sci. Total Environ..

[14] Xiao S., Li M., Chen B., Zhang Q., Wang L. (2023). Understanding the pavement texture evolution of RIOH Track using multi-scale and spatiotemporal analysis. Tribol. Int..

[15] Hu J., Ma T., Zhu Y., Zhou X., Huang X. (2021). A feasibility study exploring limestone in porous asphalt concrete: Performance evaluation and Superpave compaction characteristics. Constr. Build. Mater..

[16] Suddeepong A., Buritatum A., Dasdawan S., Horpibulsuk S., Chinkulkijniwat A. (2023). Mechanical performance of porous asphalt concrete incorporating bottom ash as fine aggregate. J. Mater. Civ. Eng..

[17] Li J., Wu Y., Qiu H., Zhang L., Zhao R. (2022). Study on interlayer bonding treatment technology of double-layer drainage asphalt pavement. Proceedings of the 2022 International Conference on Optoelectronic Information and Functional Materials (OIFM 2022).

[18] Li B., Zhao H., Zhou J., Yao T., Zhang Y. (2024). Investigation on sound absorption coefficients of porous asphalt concrete under different clogging conditions. Constr. Build. Mater..

[19] Zhang Z., Qiao Y., Giustozzi F. (2024). Investigation of the effect of sediment clogging on the hydraulic conductivity of porous asphalt mixes using CFD and DEM methods. Constr. Build. Mater..

[20] Sousa M., Dinis Almeida M., Fael C., Bentes I. (2024). Permeable asphalt pavements (PAP): Benefits, clogging factors and methods for evaluation and maintenance—A review. Materials.

[21] Kawano K., Shire T., O’Sullivan C. (2018). Coupled particle-fluid simulation of the initiation of suffusion. Soils Found..

[22] (2011). Standard Test Methods of Bitumen and Bituminous Mixtures for Highway Engineering.

[23] (2005). Standard Test Methods of Aggregate for Highway Engineering.

[24] Li B., Yang Z., Zhu X., Yao T., Guo F., Zhang Y. (2024). Correlating microscopic pore characteristics with permeability coefficient in porous asphalt concrete: An X-ray CT-based approach. Road Mater. Pavement Des..

[25] Li J., Han X., Li X., Diao H., He Z. (2024). Investigation of aggregate gradation on air voids distribution in porous asphalt concrete using X-ray CT scanning images. Case Stud. Constr. Mater..

[26] Liu T., Li Y., Chen Z., Zhang J., Lyu L., Pei J. (2024). Damage evolution in asphalt mixtures based on in-situ CT scanning. Constr. Build. Mater..

[27] Li B., Liu Z., Zhou J., Li X., Yao T., Zhang Y. (2024). Experimental and statistical study on sound absorption coefficient of porous asphalt concrete considering mesoscopic pore parameters. Constr. Build. Mater..

[28] Li B., Zhang Y., Wei D., Yao T., Hu Y., Dou H. (2025). Evolution of clogging of porous asphalt concrete in the seepage process through integration of computer tomography, computational fluid dynamics, and discrete element method. Comput.-Aided Civ. Infrastruct. Eng..

[29] Zhu X., Han Y., Li B., Tian B. (2024). Effect of clogging and cleaning on the air void microstructure of porous asphalt concrete. J. Mater. Civ. Eng..

[30] Akhtar M.N., Al-Shamrani A.M., Jameel M., KhanN A., Ibrahim Z., Akhtar J.N. (2021). Stability and permeability characteristics of porous asphalt pavement: An experimental case study. Case Stud. Constr. Mater..

[31] Jiang W., Sha A., Xiao J. (2015). Experimental study on relationships among composition, microscopic void features, and performance of porous asphalt concrete. J. Mater. Civ. Eng..

